# Saponins from *Sanguisorba officinalis* Improve Hematopoiesis by Promoting Survival through FAK and Erk1/2 Activation and Modulating Cytokine Production in Bone Marrow

**DOI:** 10.3389/fphar.2017.00130

**Published:** 2017-03-16

**Authors:** Xin Chen, Bogang Li, Yue Gao, Jianxin Ji, Zhongliu Wu, Shuang Chen

**Affiliations:** ^1^Chengdu Institute of Biology, Chinese Academy of Sciences (CAS)Sichuan, China; ^2^Graduate School, University of Chinese Academy of Sciences (CAS)Beijing, China; ^3^Di Ao Pharmaceutical GroupSichuan, China; ^4^Institute of Radiation Medicine, Academy of Military Medical SciencesBeijing, China

**Keywords:** myelosuppression, hematopoiesis, *Sanguisorba officinalis* L., saponin, Ziyuglycoside I, Ziyuglycoside II, apoptosis, cytokine

## Abstract

Radix Sanguisorbae, the root of *Sanguisorba officinalis* L. is used as traditional Chinese medicine. In recent decades, it has been reported to be clinically effective against myelosuppression induced by chemotherapy and/ or radiotherapy. However, the underlining mechanism has not been well studied. In this work, we evaluated the hematopoietic effect of total saponins from *S. officinalis* L. on myelosuppressive mice induced by cyclophosphamide and by^60^Co-γ-irradiation and confirmed the therapeutic effect. Then, we found total saponins and their characteristic constituents Ziyuglycoside I and Ziyuglycoside II can inhibit apoptosis of TF-1 cells caused by cytokine deprivation, and promote survival of mouse bone marrow nuclear cells through focal adhesion kinase (FAK) and extracellular signal-regulated kinase 1/2 (Erk1/2) activation *in vitro*. In addition, they can down-regulate macrophage inflammatory protein 2 (MIP-2), platelet factor 4 (PF4) and P-selectin secretion, which are reported to be suppressive to hematopoiesis, both *in vitro* and *in vivo*. These results suggest that promotion of survival through FAK and Erk1/2 activation and inhibition of suppressive cytokines in the bone marrow is likely to be the pharmacological mechanism underlying the hematopoietic effect of saponins from *S. officinalis* L.

## Introduction

Patients receiving chemotherapeutic agents and/or ionizing radiation often experience varying degrees of myelosuppression, characterized by the disruption of hematopoietic activity (Barreto et al., 2014). Hematopoiesis is the production of various types of mature blood cells from hematopoietic stem cells within the bone marrow. Antineoplastic agents display an indiscriminate toxicity to tumor cells and rapidly proliferating hematopoietic progenitor cells (Parchment et al., 1998). The anti-tumor therapy also causes myelotoxicity indirectly by affecting the bone marrow microenvironment including stromal cells and hematopoietic regulators in the bone marrow. Leukocytes have the shortest half-life in circulation and a decrease in leukocytes count (neutropenia) is a frequently occurring consequence of myelosuppression. Profound myelotoxicity will lead to blood losing events or severe infection presented with sepsis and febrile neutropenia. Cancer treatment-induced hematologic toxicities still are the main reasons of mortality and morbidity throughout the therapy of cancer (Wang et al., 2006).

Granulocyte colony-stimulating factor is currently used as an adjunct to chemotherapy for alleviating neutropenia. However, there are some commonly reported side-effects associated with administration of G-CSF including bone pain, flushing and nausea (American Society of Clinical Oncology, 1994; Khoury et al., 2000; Ozer et al., 2000).

Radix Sanguisorbae, the root of *Sanguisorba officinalis* L., is used to alleviate neutropenia in recent decades in China (Liang et al., 2008). Radix Sanguisorbae is a well known herbal medicine in China, Japan and Korea. It has hemostatic and astringent properties (East, 1955), and is traditionally used in clinical practice for the treatment of scalds-and-burns, bleeding, diarrhea, duodenal ulcers, and chronic intestinal infections (Zhang L. et al., 2012). The anti-inflammatory (Yu et al., 2011; Ravipati et al., 2012), anti-infection (Liang et al., 2013; Liu et al., 2014), antioxidant (Ravipati et al., 2012; Zhang L. et al., 2012), anti-cancer (Cai et al., 2012; Choi et al., 2012), anti-allergic (Kim et al., 2002; Park et al., 2004), anti-wrinkle (Kim et al., 2008), and neuroprotective (Ban et al., 2008; Nguyen et al., 2008) activities of extracts of *S. officinalis* L. roots or their bioactive constituents have been reported. The bioactive constituents in Radix Sanguisorbae include tannin, flavone, steroid, and saponin compounds (Xia et al., 2009). Ziyuglycoside I and Ziyuglycoside II are the characteristic constituents of saponin components extracted from Radix Sanguisorbae. In additional to *S. officinalis* L. roots which are used as Traditional Chinese Medicine in clinic, a recent work by Szejk et al. (2017) reported the radioprotective effects of the polyphenolic glycoconjugates from flowers of *S. officinalis* L.

**FIGURE 1:**
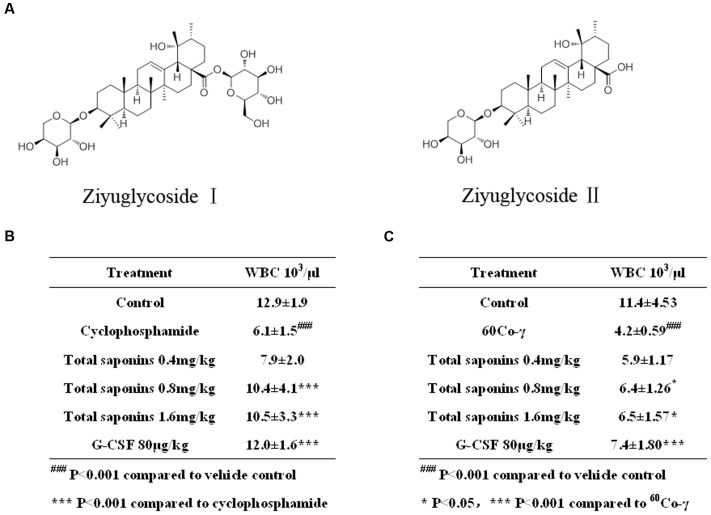
**Total saponins enhanced hematopoietic recovery in myelosuppression mouse model.** **(A)** Ziyuglycoside I and ziyuglycoside II. **(B)** Peripheral blood WBC analysis on Day 7 after the injection of 100 mg/kg cyclophosphamide. ^###^*p* < 0.001 compared to vehicle control, ^∗∗∗^*p* < 0.001 compared to cyclophosphamide. **(C)** Peripheral blood WBC analysis on Day 13 after the 3.5Gy ^60^Co-γ irradiation. ^###^*p* < 0.001 compared to vehicle control, ^∗^*p* < 0.05 ^∗∗∗^*p* < 0.001 compared to ^60^Co-γ. Data were presented as mean ± SD (*n* = 13).

The clinical reports in recent decades demonstrated the prophylactic effect of Radix Sanguisorbae on myelosuppression induced by chemotherapy (Li et al., 2005) or radiotherapy (Zhang R. et al., 2012). The therapeutic effect of Radix Sanguisorbae against myelosuppression in patients was also reported (Fu, 2014; Jiang et al., 2015). Moreover, no severe side effects have been reported. The above clinical reports suggest that Radix Sanguisorbae can enhance hematopoietic recovery. Recent pharmacological studies in mice demonstrated that saponin components from Radix Sanguisorbae are major active constituents possessing hematopoietic effect. Administration of the saponins increased the number of bone marrow cells, leucocytes, erythrocytes, and platelets of cyclophosphamide-induced myelosuppressive mice (Gao et al., 2006). In the present study, we found that the total saponins from *S. officinalis* L. roots can ameliorate myelosuppression induced by cyclophosphamide and^60^Co-γ-irradiation on mice. Ziyuglycoside I and Ziyuglycoside II (**Figure 1A**) are the characteristic constituents of total saponins exacted from *S. officinalis* L. roots (Cheng and Cao, 1992). We investigated the effect of the total saponins, Ziyuglycoside I and Ziyuglycoside II on hematopoietic cells and the secretion of cytokines by using *in vitro* and *in vivo* assays. Our findings suggest that the activation of FAK and Erk1/2, and modulation of cytokine production may be the possible pharmacological mechanisms of their hematopoietic activity.

## Materials and Methods

### Reagents

Ziyuglycoside I and Ziyuglycoside II was supplied by Chengdu Herbpurify Co., Ltd (China), dissolved in DMSO at the concentration of 100 mg/ml. RPMI Medium 1640 and FBS were obtained from Thermo Fisher Scientific Inc. Cell Titer-Glo Luminescent Cell Viability Assay and Caspase-Glo 3/7 assay system were purchased from Promega Biotech Co., Ltd. The recombinant human G-CSF, (GM-CSF, EPO, and rmSCF), GM-CSF, interleukin-3 (rmIL-3) were bought from ProSpec-Tany Techno Gene Ltd. The inhibitor of FAK (PF 573228) and Erk1/2 (FR 180204) were obtained from Selleckchem.

### Total Saponins Extraction and Isolation

The total saponins of Radix Sanguisorbae were prepared as follows. In brief, air-dried roots of *Sanguisorba officinalis* L. were purchased in the local market in Chengdu, China. One kilogram of air-dried roots were sliced, and then reflux-extracted with ethanol. After filtration, the ethanol extract was diluted with 1.5 volume of distilled water and PH was adjusted to 10.5 by adding the alkaline solution (KOH) under stirring. After centrifugation, the supernatant was subjected to HPD100 macroporous absorption resin and eluted using acetone. The elution was evaporated under vacuum to yield the total saponins from Radix Sanguisorbae. In the extract of total saponins, the content of Ziyuglycoside I was 45∼50% (16.35∼18.17 mg/g dry weight), and content of Ziyuglycoside II was 6∼8% (2.18∼2.91 mg/g dry weight).

### Animal Experiments

BALB/c mice, 6- to 8-week-old, were purchase from Da-suo Bio-technology Limited (Chengdu, Sichuan, China). Kunming mice, weighting 18–22 g, were supplied by Experimental Animal Center of the Academy of Military Medical Sciences. Mice were housed under controlled temperature and humidity and a lighting cycle of 12 h/day. The mice were fed with untreated tap water and commercial mice chow.

Cyclophosphamide was used to induce myelosuppression in mice. The total saponins dissolved in saline were administered orally for 14 day. Kunming mice were administrated (i.p. injection) of cyclophosphamide at 100 mg/kg on Day 3 to 5. On Day 12, peripheral blood was collected from tail veins for determination of WBC.

Irradiation was used to induce myelosuppression in mice. A cobalt-60 γ-radiation source was used. Kunming mice were placed in well-ventilated boxes and were irradiated of the whole body at a single dose of 3.5Gy ^60^Co-γ irradiation on Day 1. Rate of irradiation used was 1.27Gy per minute and the distance between the source of irradiation and mice was 4 meters. Then total saponins were administered orally for 14 day. Peripheral blood WBC was analyzed on Day 13.

### Cell Culture

Human bone marrow TF-1 cells (ATCC CRL-2003) were maintained in RPMI 1640 supplemented with 10% FBS and 2 ng/mL of recombinant human GM-CSF until use. TF-1 cells were used between passage 6 and 15.

Mouse BMNCs were harvested from femurs of 6- to 8-week-old BLAB/c mice. Femora were dissected free of muscle and connective tissue, and cut at one end under sterile conditions. The marrow plugs were forced out with RPMI1640 (Invitrogen) medium supplemented with 20% FBS, and filtered through a 75-μM filter (Millipore). Then cells were rinsed with RPMI1640 medium and red blood cells were lysed with Tris-NH_4_Cl solution. The erythrocytes- depleted BMNCs were kept in RPMI1640 medium with 20% FBS for further use.

Mouse BMSCs were isolated by their adherence to plastic. Briefly bone marrow cells were collected by flushing the femurs with RPMI1640 medium supplemented with 20% FBS. The marrow plugs were filtered through a 75-μM filter (Millipore). Cells were plated at a density of 2 × 10^6^cells in 6-well plates, and the non-adherent cell population was removed after 72 h. The medium was subsequently replaced twice weekly. The cells were grown for 7–10 days until almost confluent.

### Cell Viability Assay

Cells were seeded in 96-well plates and subjected to the saponins treatment for indicated time periods. The viability of cells was determined by ATP luminescent assay (Promega). In brief, equilibrate the plate at room temperature for approximately 30 min, then add a volume of CellTiter-Glo reagent equal to the volume of cell culture medium present in each well. Mix contents for 2 min on an orbital shaker to induce cell lysis, and allow the plate to incubate at room temperature for 10 min then record RLU.

%Growth =(RLU sample-RLU control)/RLU control×100.

### Caspase3/7 Activity Assay

TF-1 cells were maintained in culture medium free of GM-CSF 2 days before saponins treatment. Then cells were seeded at a density of 1.5 × 10^4^ cells in 96-well plates in the presence or absence of saponins for 48 h. The activities of caspase-3 and -7 were detected by luminescent assay (Promega). Briefly equilibrate the plate at room temperature for approximately 30 min, then add 100 μL Caspase-Glo 3/7 reagent to each well of cells in equal volume of culture medium. Mix contents for 30 s, and incubate at room temperature for 1 h then record luminescence.

### Western Blot Analysis

TF-1 cells were seeded at a density of 5 × 10^6^ cells in 6-well plates, and treated with saponins or DMSO over a period of 24 h. To measure the phosphorylation of Erk1/2 protein, mouse BMNCs were seeded at a density of 1 × 10^7^ cells in 6-well plates and treated with or without saponins for different times. Whole cell protein was extracted using a Total Protein Extraction Kit (CWBiotech). Protein concentration in the supernatant was determined by a BCA protein assay (Bio-Rad). Equal amounts of protein were loaded on an SDS-poly-acrylamide gel and transferred to a PVDF membrane (Millipore) before blocking with 5% nonfat dried milk in PBS with 0.1% Tween-20. Primary and secondary antibodies were diluted in blocking solution. Primary antibodies used in this study were bcl-2, bcl-xl, Mcl-1, survivin, Bax, Bim, pErk1/2 (Cell Signaling Technology). Bound primary antibodies were detected with SuperSignal Western Blot Substrate (Thermo scientific).

### Immunoprecipitation and *In vitro* Kinase Assay

For the immunoprecipitation studies, saponins-stimulated, or unstimulated mouse BMNCs were lysed in ice-cold lysis buffer (Cell Signaling Technology) adding 1 mmol/L PMSF. Lysates were precleared with rabbit isotype control (Cell Signaling Technology) and protein G plus/protein A agarose beads (Millopore) for 1 h at 4°C. After removal of agarose beads by brief centrifugation, protein concentration in the supernatant was determined and the precleared lysates were incubated with anti-pFAK (p576/577) overnight at 4°C. The immunocomplexes were captured by incubation for 3 h at 4°C with protein G/A agarose beads. Immunoprecipitates were washed three times with phosphate-buffered saline, two times with kinase reaction buffer A (Promega). Bound proteins were resuspended in 25 μL kinase assay buffer, and phosphatase activity of immunoprecipitated FAK was assessed using a FAK kinase assay (Promega) following the manufacturer’s instructions. FAK activity was normalized with respect to the total protein content of cell lysates.

### Cytokine Arrays

Mice were treated with total saponins and/or cyclophosphamide as described in Section “Animal Experiments”. Mice were killed by cervical dislocation and the femurs were dissected free of muscle and tendons, and then marrow was extruded by flushing the shaft of the bone with 0.5 ml of RMPI1640 supplemented with 20% FBS. The resulting cell suspension was filtered through a 75-μM filter and particulates were removed by centrifugation. The mouse cytokine antibody array (Ray Biotech) was used to study the expression profile of cytokine proteins in the clarified supernatant following the manufacturer’s instructions.

Confluent primary mouse BMSCs were seeded at a density of 25 × 10^4^ cells and grown in 24-well plates in a final volume of 1 mL culture medium per well. After 24 h, the cells were treated with total saponins or vehicle control over a period of 24 h. The cytokines in the cell culture supernatant were evaluated using the cytokine antibody array as mentioned above.

### Cytokine ELISA

Confluent primary mouse BMSCs were seeded at a density of 8 × 10^4^ cells and grown in flatbottomed96-well plates in a final volume of 200 μL culture medium per well. After 24 h, the cells were treated with saponins or DMSO over a period of 24 h. The culture supernatant of control and treated cells were collected for measurement by centrifugation to remove particulates. The level of PF4, MIP-2, and sP-Selectin in supernatant was measured using the enzyme-linked immunoassay kit (R&D Systems) according to the protocol of the manufacturer. The results were normalized to the cell number of BMSCs.

### Co-culture

Confluent primary mouse BMSCs were seeded in the 24-well plates at a density of 25 × 10^4^ cells in a final volume of 1 mL culture medium per well. After 24 h, the cells were treated with saponins or DMSO over a period of 6 h. Then fresh medium was added following removal of drugs. Mouse BMNCs were seeded into the cell culture insert (Falcon, BD Bioscience) at a concentration of 7 × 10^5^ cells, and co-cultured with the pretreated mouse BMSCs monolayer. Cells were harvested after 48h, the viability of cells was assessed using ATP luminescent assay as described above.

### Statistical Analysis

Values were given as mean ± standard deviation of at least three independent experimental replications. Comparisons between mean values were made using one way ANOVA with a Fisher’s LSD test, and *p* < 0.05 was determined to indicate statistical significance.

## Result

### Total Saponins from *S. officinalis* L. Enhanced Hematopoietic Recovery in Myelosuppression Mouse Model

Compared to the vehicle control group, the injection of cyclophosphamide and irradiation of ^60^Co-γ decreased WBC dramatically (*p* < 0.001), as indicated in **Figure 1**. The hypodermic injection of rhG-CSF alleviated neutropenia significantly (*p* < 0.001), compared to the group treated with cyclophosphamide or ^60^Co-γ alone. The oral administration of total saponins either 1.6 mg/kg or 0.8 mg/kg for 7 days following chemotherapy increased WBC significantly (*p* < 0.001), compared to the group treated with cyclophosphamide alone (**Figure 1B**). Administration of total saponins at 1.6, 0.8, and 0.4 mg/kg increased WBC of cyclophosphamide treated mice from 6.1 ± 1.5 × 10^3^/μL to 10.5 ± 3.3, 10.4 ± 4.1, and 7.9 ± 2.0 × 10^3^/μL, respectively. The oral administration of total saponins either 1.6 mg/kg or 0.8 mg/kg for 13 days following radiotherapy alleviated neutropenia significantly (*p* < 0.05) (**Figure 1C**), compared to the group treated with ^60^Co-γ exposure alone. Administration of total saponins at 1.6, 0.8, and 0.4 mg/kg increased WBC of ^60^Co-γ irradiated mice from 4.2 ± 0.59 × 10^3^/μL to 6.5 ± 1.57, 6.4 ± 1.26 and 5.9 ± 1.17 × 10^3^/μL, respectively.

### Saponins from *S. officinalis* L. Promoted Survival of Bone Marrow Cells *In vitro*

We investigated the effect of total saponins from *S. officinalis* L. roots and their characteristic constituents Ziyuglycoside I and Ziyuglycoside II on the viability of bone marrow cells *in vitro*. Bone marrow hematopoietic cells are hematopoietic cytokine dependent in the *in vitro* culture and we performed the assay in the presence and absence of hematopoietic cytokines.

**FIGURE 2:**
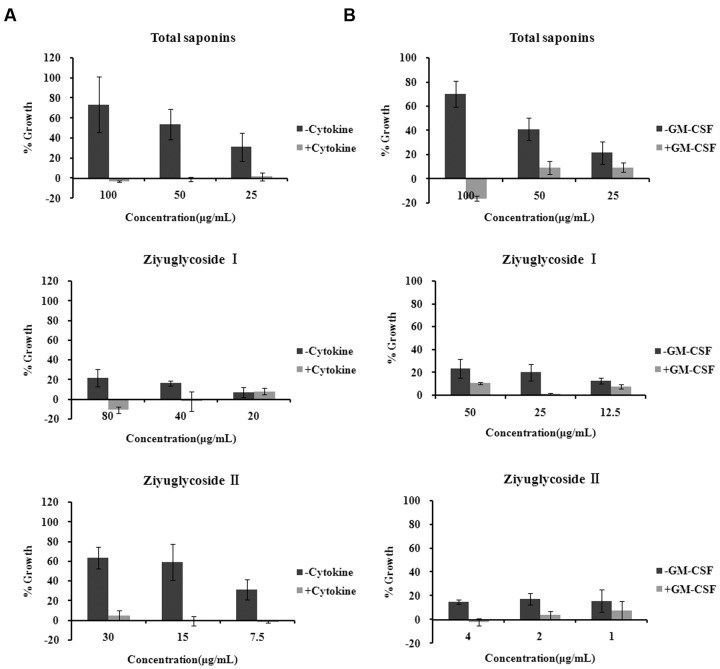
**Saponins from *Sanguisorba officinalis* L. promoted survival of bone marrow cells.** **(A)** Mouse BMN cells were maintained in medium free of cytokine or complete medium (containing 50 ng/mL rmSCF, 10 ng/mL rmIL-3, 10 ng/mL rmGM-CSF, 3 U/mL Epo). Mouse BMNCs were seeded at a density of 7 × 10^5^ cells in 96-well plates in the presence or absence of saponins for 6 days. **(B)** TF-1 cells were cultured in medium free of GM-CSF 2 days before saponins treatment or complete medium (containing 2 ng/mL rhGM-CSF). TF-1 cells were seeded at a density of 1.5 × 10^4^ cells in 96-well plates and stimulated with saponins for 48 h. All experiments were repeated at least three times. Data were presented as mean ± SD.

Mouse BMNCs were prepared and cultured with saponins for 6 days. We found that in the cytokine free group, treatment with total saponins, Ziyuglycoside I and Ziyuglycoside II resulted in a dose-dependent increase in cell viability by up to 73% (±28%), 22% (±9%), and 63% (±11%), respectively, as compared to vehicle-treated control cells, whereas in the presence of cytokines in the medium (a mixture of GM-CSF, SCF, IL-3, EPO, which are commonly used to support hematopoietic progenitor cells in the *in vitro* culture), treatment by saponins exhibited little improvement in cell viability as compared to vehicle-treated control (less than10%) (**Figure 2A**).

TF-1 is a bone marrow-derived human hematopoietic progenitor cell line and we also used them to evaluate the effect of saponins. TF-1 cells are cytokine dependent and are normally cultured in medium supplemented with GM-CSF. Under such culture condition, treatment by saponins (48 h) had little effect on cell viability as compared to vehicle-treated control (less than10%). In the cytokine free group cells were depleted of GM-CSF 48h before saponins stimulation. Treatment of cytokine-deprived TF-1 cells with total saponins, Ziyuglycoside I and Ziyuglycoside II (48 h) induced up to a 70% (±11%), 24% (±8%), and17% (±5%) increase in cell viability, respectively, as compared to vehicle-treated control (**Figure 2B**). These data demonstrated that total saponins from *S. officinalis* L. roots, Ziyuglycoside I and Ziyuglycoside II were able to promote survival of bone marrow cells *in vitro*.

### Saponins from *S. officinalis* L. Exerted Anti-apoptotic Effects on TF-1 Cells

**FIGURE 3:**
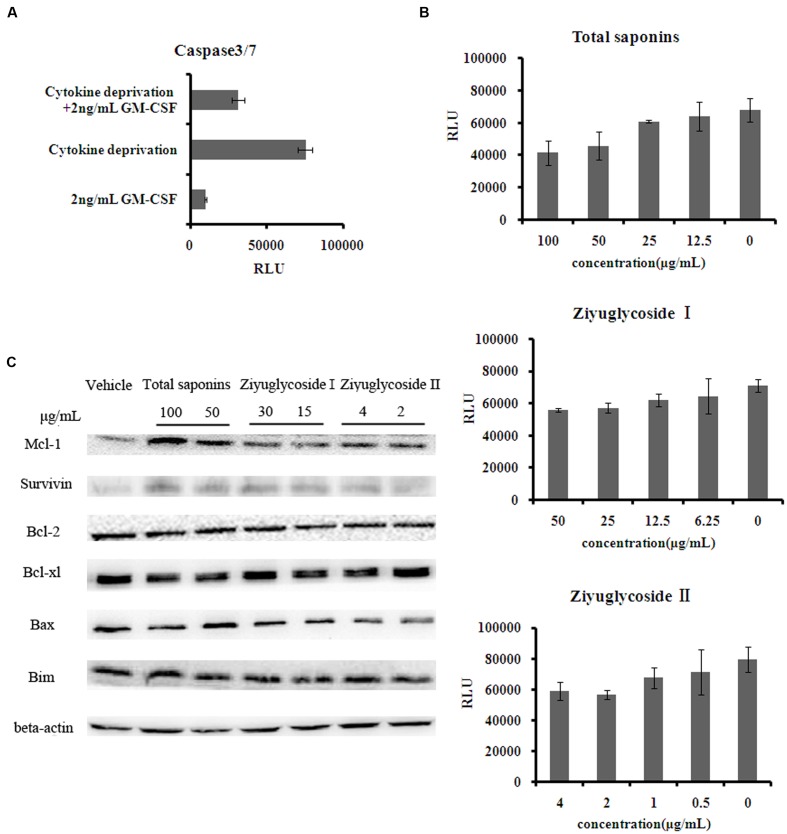
**Saponins from *S. officinalis* L. exerted anti-apoptotic effects on TF-1 Cells.** **(A–B)** The activity of caspase3/7 in TF-1 cells. Assays were done in triplicate. Mean values with standard deviations are shown. **(C)** Change of protein expression patterns in TF-1 cells. Cells were cultured in medium free of GM-CSF for 2 days. Then cells were treated with saponins or vehicle for 48 h. Representative results of three independent experiments are shown.

As shown above, saponins from *S. officinalis* L. roots enhanced the survival of cytokine-deprived TF-1 cells. It has been reported that cytokine deprivation in TF-1 cells induced apoptosis (Lin et al., 2007). Consequently, we tested the anti-apoptotic effect of the saponins, by measuring the activity of caspase-3 and -7 which are downstream effector caspases executing apoptosis. Consistent with other reports, GM-CSF withdrawal for 48h increased caspase-3 and -7 activities by 7 times the complete medium control (**Figure 3A**). The high levels of caspase-3 and -7 activities induced by cytokine deprivation were reduced by saponins treatment in a dose-dependent manner. Treating cytokine-deprived TF-1 cells with total saponins, Ziyuglycoside I, and Ziyuglycoside II (48 h) inhibited caspase-3 and -7 activities up to 39% (±11%), 27% (±6%), and 26% (±8%), respectively, as compared to vehicle-treated control (**Figure 3B**). Then, we detected the protein level of anti-apoptotic protein survivin and several Bcl-2 family members including anti-apoptotic bcl-2, bcl-xl, Mcl-1, and pro-apoptotic Bim and Bax. We found that saponins treatments increased the protein expression of anti-apoptotic Mcl-1 and survivin (**Figure 3C**) in GM-CSF deprived TF-1 cells and other proteins we tested were not affected. These data suggested that up-regulation of Mcl-1 and survivin by saponins was involved in their anti-apoptosis effect on cytokine-deprived TF-1 cells.

### Activation of FAK and Erk1/2 was Involved in the Pro-survival Effect of Saponins from *S. officinalis* L. on Bone Marrow Cells

**FIGURE 4:**
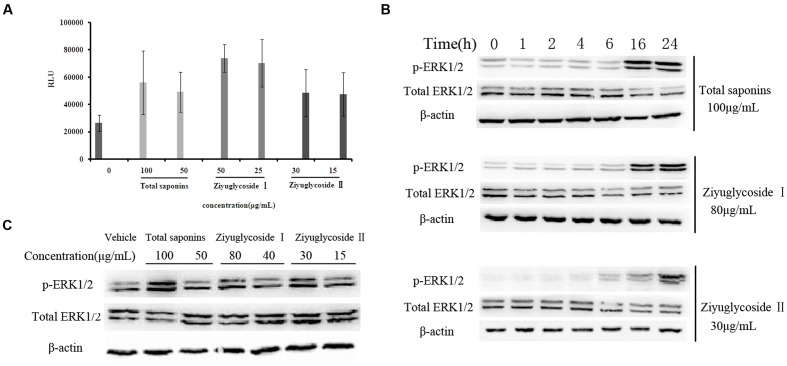
**Saponins from *S. officinalis* L. induced activation of FAK and ERK1/2.** **(A)** *In vitro* kinase assay for tyrosine phosphorylation-associated FAK kinase activity. The assay was performed in triplicate. Data were presented as mean ± SD. **(B–C)** Western blotting analysis for ERK1/2 phosphorylation. The time point was 16h in **(C)**. Representative results of three independent experiments are shown.

From our previous work (data not shown), we speculated that total saponins from *S. officinalis* L. roots could activate FAK and Erk1/2. In this study, we confirmed that treatment of total saponins, Ziyuglycoside I and Ziyuglycoside II elevated activity of FAK and Erk1/2 on bone marrow cells. As shown in **Figure 4A**, we measured kinase activity of FAK by *in vitro* kinase assay after immunoprecipitated with anti-pFAK (p576/577) antibody and we found that the tyrosine phosphorylation-associated FAK kinase activity was enhanced at 2 h after saponins treatment in a dose-dependent manner. Treatment with total saponins, Ziyuglycoside I and Ziyuglycoside II induced up to a 112, 180, and 83% increase in pFAK activity, respectively, as compared to vehicle-treated control. As shown in **Figure 4**, western blot analysis using anti-pERK1/2 antibody showed that levels of phosphorylation of Erk1/2 increased after saponins stimulation at later time points (**Figure 4B**, 16 h∼24 h for total saponins and Ziyuglycoside I, and 6 h∼24 h for Ziyuglycoside II) in a dose dependent manner (**Figure 4C**).

**FIGURE 5:**
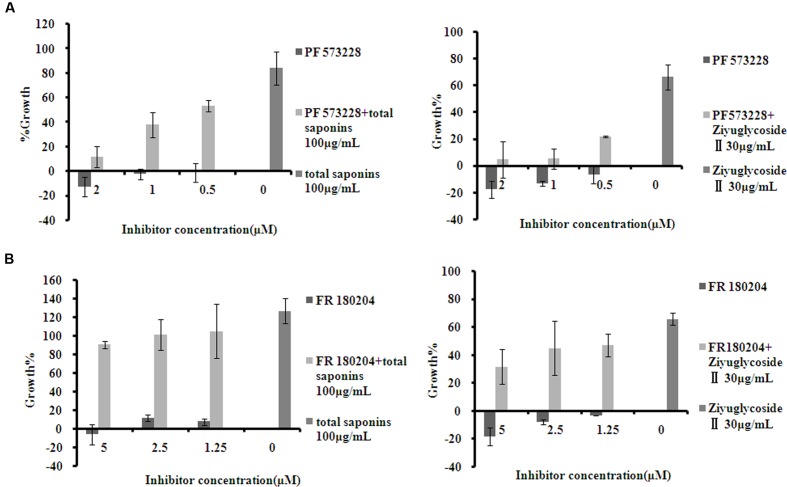
**The inhibitor of FAK and Erk1/2 suppressed the pro-survival effect of saponins on bone marrow cells.** **(A)** Viability of mouse BMN cells treated with PF 573228 (inhibitor of FAK), either alone or in combination with saponins. **(B)** Viability of mouse BMN cells treated with FR 180204 (inhibitor of Erk1/2), either alone or in combination with saponins. The experiment was repeated at least three times. Data were presented as mean ± SD.

To investigate whether the pro-survival effect of saponins was mediated through FAK and Erk1/2 activation, the inhibitor of FAK (PF 573228) and Erk1/2 (FR 180204) were used in combination with the saponins. As shown in **Figure 5**, different dose of PF 573228 (**Figure 5A**) or FR 180204 (**Figure 5B**) alone had no obvious influence on cell viability, while both of them effectively suppressed the pro-survival effect of saponins on bone marrow cells in a dose-dependent manner. When total saponins (100 μg/mL) was used in combination with PF 573228 (2, 1, 0.5 μM), the increase in cell viability reduced from 84 to 12%, 38 and 53%, respectively. While FR 180204 (5, 2.5, 1.25 μM) was used in combination with total saponins (100 μg/mL), the increase in cell viability reduced from 126 to 90%, 101 and 105%, respectively. When Ziyuglycoside II (30 μg/mL) was used in combination with PF 573228 (2, 1, 0.5 μM), the increase in cell viability reduced from 66 to 4%, 5 and 22%, respectively. While FR 180204 (5, 2.5, 1.25 μM) was used in combination with total saponins (30 μg/mL), the increase in cell viability reduced from 66 to 32%, 45 and 47%, respectively.

### Saponins from *S. officinalis* L. Reduced Suppressive Cytokines in Bone Marrow Microenvironment

Cytokines in the bone marrow microenvironment play vital roles in the regulation of hematopoiesis. So, we investigated the effect of total saponins from *S. officinalis* L. roots on bone marrow microenvironment *in vivo* and *in vitro* by performing a mouse cytokine antibody array. As a model of myelosuppression, mice were injected with cyclophosphamide, and then, were co-administrated with either total saponins or vehicle as described in Section “Animal Experiments”. The mouse bone marrow supernatant fluid were collected and measured for cytokine expression. Compared to the myelosuppression group, Levels of seven cytokines (MIP-1-γ, MIP-2, PF4, RANTES, sTNF RI, sTNF RII, and P-selectin) reduced in the total saponins treatment group compared to the control myelosuppression group. BMSCs secrete various cytokines into the bone marrow. So, we also isolated BMSCs and measured their cytokine expression after total saponins stimulation. Level of thirteen cytokines (CXCL16, IFN-γ, IL-3, IL-4, IL-6, IL-12-p70, MIG, MIP-2, MIP-3α, PF4, P-selectin, VCAM-1, VEGF) decreased and two (BLC, Eotaxin-2) increased in cell culture supernatant of BMSCs after total saponins treatment (treatment/ control >1.2 or <0.8).

**FIGURE 6:**
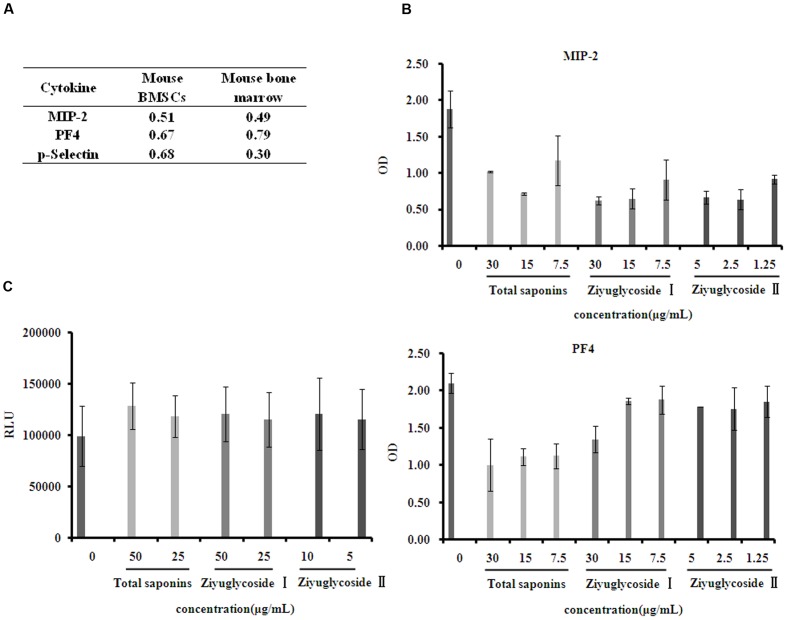
**Saponins from *S. officinalis* L. inhibited suppressive cytokine production.** **(A)** Mouse cytokine antibody array showing fold change in gene expression patterns of MIP-2, PF4, and P-selectin in bone marrow and conditioned medium of BMSCs. To collect sample of bone marrow, mice were treated with total saponins and cyclophosphamide as described in Section “Animal Experiment”, and mouse bone marrow supernatant fluid was collected as mentioned in Section “Cytokine Arrays”, and cyclophosphamide treatment alone was used as control (*n* = 8). The conditioned medium of BMSCs was collected as mentioned in Section “Cytokine Arrays”, and vehicle treatment was used as control. The fold change value is calculated by dividing the data from treatment with control. **(B)** ELISA analysis for MIP-2 and PF4 in cell culture supernatant of mouse BMSCs. **(C)** Viability of mouse BMN cells co-cultured with BMSCs pretreated with saponins. The experiment was repeated at least three times. Data were presented as mean ± SD.

The expression of MIP-2, PF4, and P-selectin reduced after total saponins treatment both in the *in vivo* and *in vitro* study (**Figure 6A**). According to previous reports, these three cytokines are negative regulators of hematopoiesis. To validate the array data, we measured their protein levels on primary mouse BMSCs treated with different dose of total saponins, Ziyuglycoside I and Ziyuglycoside II by performing ELISA assay. Compared with untreated cells, saponins treatment for 24 h reduced the protein level of MIP-2 and PF4 in cell culture supernatant in a dose-dependent manner (**Figure 6B**). Treatment with total saponins, Ziyuglycoside I and Ziyuglycoside II resulted in up to 62, 67, and 65% inhibition of MIP-2 protein expression, respectively, and 52, 36, and 15% reduction of PF4 protein level, respectively, as compared to vehicle-treated control. We could not detect P-selectin in the cell culture supernatant using ELISA method. This may be because the content of P-selectin in cell culture supernatant may be under the minimum detectable dose of our method.

To assess how bone marrow cell growth was affected by the reduction of hematopoiesis negative regulators caused by saponins treatment, we performed co-culture experiments using mouse BMSCs and primary bone marrow cells (BMNCs). Mouse BMSCs were pretreated with different dose of total saponins, Ziyuglycoside I and Ziyuglycoside II, and then co-cultured with BMNCs in the upper inserts. Compared with untreated control, saponins-pretreatment increased the viability of BMNCs co-cultured (**Figure 6C**). Pretreatment with total saponins, Ziyuglycoside I and Ziyuglycoside II (6 h) induced up to a 29, 21, and 21% increase in cell viability, respectively, as compared to vehicle-treated control. These results indicated that saponins of *S. officinalis* L. roots could improve the survival of BMNCs by reducing secretion of hematopoiesis negative cytokines from BMSCs.

## Discussion

The extract of *S. officinalis* L. roots has been reported to have therapeutic effect on chemotherapy- and radiotherapy-induced neutropenia in clinic in China. However, the mechanism of its therapeutic effect on neutropenia has not been reported yet. In this study, firstly we assessed the effect of total saponins from *S. officinalis* L. roots on myelosuppression mouse models and demonstrated that total saponins accelerated the hematopoietic restoration after injury induced by cyclophosphamide and^60^Co-γ-irradiation. This result is consistent with the clinical findings of the therapeutic effects of *S. officinalis* L. roots on neutropenia.

Ziyuglycoside I and ziyuglycoside II are the characteristic constituents of total saponins from *S. officinalis* L. roots. To further reveal the role of total saponins in the regulation of hematopoiesis, we then investigated the effect of total saponins and their characteristic constituents on the growth of bone marrow cells. We used primary bone marrow cells (BMNCs) and cytokine-dependent TF-1, which is a human hematopoietic progenitor cell line with CD34^+^ phenotype (Civini et al., 2013), to carry out the study. We found that in cytokine-free medium total saponins, Ziyuglycoside I and Ziyuglycoside II greatly increased cell viability compared with the vehicle-treated control. Using cytokine-deprived TF-1 cells as apoptosis model, we found that total saponins, Ziyuglycoside I and Ziyuglycoside II can reduce apoptosis caused by cytokine deprivation and anti-apoptotic proteins Mcl-1 and survivin were involved in the anti-apoptotic effect of saponins. We further investigated pathways involved in the pro-survival effect of saponins on bone marrow cells.

Focal adhesion kinase is a nonreceptor protein tyrosine kinase, transducing signals from integrins, some cytokines, and growth factor receptors. It is expressed in many hematopoietic cells, including granulocyte macrophage progenitor cells, erythroid progenitors and lymphoid cells, etc. (Choi et al., 1993; Kume et al., 1997). Activation of integrins or cytokine receptors interacted induces rapid phosphorylation on tyrosine residue 397 of FAK and subsequent phosphorylation of tyrosine residue 576/577 in activation loop of FAK, which results in full catalytic activity (Lietha et al., 2007). To test whether the saponins from *S. officinalis* L. roots could activate FAK, we immunoprecipitated phospho-FAK (Tyr576/577) from both vehicle- and saponins-treated mouse BMNCs lysates, followed by *in vitro* kinase assay, and found that saponins treatment increased the kinase activity of FAK. It has been reported that FAK play a crucial role in regulating stress hematopoiesis (Vemula et al., 2010) and hematopoietic cell lodgment and lineage development (Glodek et al., 2007). In the current study, we found that saponins induced survival enhancement of bone marrow cells could be blocked by inhibition of FAK activity using the FAK inhibitor PF 573228. These results indicated the involvement of FAK activation in the improvement of cell survival after saponins treatment.

Extracellular signal-regulated kinase are serine/threonine kinases that play critical roles in regulation of cell growth, proliferation and survival (Geest and Coffer, 2009). It has been demonstrated that ERK1/2 signaling is involved in the normal hematopoiesis (Staser et al., 2013), and is important in maintaining hematopoietic homeostasis (Chung and Kondo, 2011). We performed Western blot assay to detect the phosphorylation of ERK1/2, and found that saponins treatment up-regulated the phospho-ERK. In addition, the survival enhancement of bone marrow cells in response to saponins was blocked by ERK1/2 inhibitor FR 180204, indicating that saponins may improve the survival of bone marrow cells through ERK1/2 activation.

The bone marrow microenvironment plays a vital role in hematopoiesis. Hematopoietic growth factors, cytokines and chemokines in the microenvironment secreted by BMSCs regulate the development of hematopoietic cells (Majka et al., 2001). We monitored the levels of 62 cytokines in cultured mouse BMSCs treated with total saponins, using a mouse cytokine antibody array. The levels of cytokines in bone marrow of mice were determined using the same array. Reduced expression of PF4, MIP-2, and P-selectin was observed both in the conditioned medium of total saponins-treated mouse BMSCs, and in the bone marrow of total saponins-treated myelosuppressive mice.

PF4 is a member of the C-X-C chemokine family. It is not a potent chemotaxin agent (Deuel et al., 1981; Petersen et al., 1996), but strongly inhibits hematopoiesis (Lecomte-Raclet et al., 1998). PF4 has been reported to be able to suppress colony formation of early committed myeloid progenitor cells at low concentrations in the nanomolar range *in vitro* (Broxmeyer et al., 1993), and inhibit proliferation of human CD34^+^ hematopoietic progenitor cells (Dudek et al., 2003). In the study of PF4 knockout mice and transgenic mice overexpressing human PF4, platelet count and megakaryocyte colonies were inversely associated with PF4 content, and treatment with anti-PF4 blocking antibodies accelerated the recovery of 5-Fu-induced marrow failure, especially in PF4-overexpressing mice (Lambert et al., 2007).

The myelosuppressive effects of MIP-2 and P-selectin on hematopoiesis has been described by other researchers (Broxmeyer et al., 1995; Eto et al., 2005). Mouse MIP-2 is classified as a member of the C-X-C chemokine family, and it is homolog of human MIP-2α based on the protein sequence similarity. MIP-2α has been reported to be suppressive to immature subsets of stem and progenitor cells *in vitro* (Broxmeyer et al., 1993). P-selectin is cell adhesion molecule, which is involved in the interaction between hematopoietic progenitor cells and the stromal cell in the bone marrow microenvironment. P-selectin could inhibit the proliferation of human and mouse hematopoietic progenitor cells *in vitro* (Levesque et al., 1999), and mice deficient for both P- and E-selectin presented enhanced hematopoiesis (Frenette et al., 1996).

Treatment with total saponins reduced the level of MIP-2, PF4, and P-selectin in both bone marrow of mice and cell culture supernatant of mouse BMSCs. ELISA results further validated that total saponins, Ziyuglycoside I and Ziyuglycoside II suppress the expression of MIP-2 and PF4 dose-dependently. To study whether saponins of *S. officinalis* L. roots could affect hematopoiesis by reducing secretion of suppressive cytokines, we co-cultured BMNCs with BMSCs pretreated with saponins. The result confirmed that saponins of *S. officinalis* L. roots could influence hematopoietic microenvironment by modulating cytokine production of BMSCs, and thus promoted survival of BMNCs co-cultured.

Ziyuglycoside I is the glucopyranose ester of Ziyuglycoside II. In the present study, we observed activity difference between Ziyuglycoside I and II *in vitro*. Treatment with Ziyuglycoside I (25μg/mL) induced 166% increase in pFAK kinase activity, while Ziyuglycoside II (30 μg/mL) induced 83% increase compared to vehicle-treated control (**Figure 4A**). The level of phosphorylation of Erk1/2 increased 6 h after Ziyuglycoside II (30 μg/mL) stimulation, while obvious increase of pErk1/2 could not be observed until 16 h after Ziyuglycoside I (80 μg/mL) stimulation (**Figure 4B**). The structure-activity relationship of Ziyuglycoside I and II require further studies.

Dozens of saponins have been identified in extracts of *S. officinalis* L. roots and most of them are triterpenoid saponin (Cheng and Cao, 1992; Mimaki et al., 2001; Liu et al., 2005). Ziyuglycoside I and ziyuglycoside II are characteristic constituents of *S. officinalis* L. roots and various bioactivities of them have been studied. Kim and their colleagues reported the anti-wrinkle activity of Ziyuglycoside I by increasing type I collagen expression in fibroblast cells and its application as anti-wrinkle cosmeceutical ingredient (Kim et al., 2008). Radix Sanguisorbae is traditional used as a hemostatic agent in clinic and Ziyuglycoside I was reported to have hemostatic activity by effecting α2-plasmin inhibitor (Sun et al., 2012). Ziyuglycoside I was reported to have anti-cancer effect in a breast cancer cell line through up-regulation of p53 and p21 (Zhu et al., 2016) and Ziyuglycoside II was reported to have anti-cancer activity by induction of reactive oxygen species (ROS) and ROS dependent apoptotic pathways in tumor cell lines (Zhu et al., 2014). But there is not clinical practice of using *S. officinalis* L. or its bioactive constituents as anti-cancer agents, and there is little information available about toxicological studies of saponins from *S. officinalis* L.. These reports suggested that Ziyuglycoside I and ziyuglycoside II can exert various biology effects by activating different pathways in different cells. In the present work, we found that Ziyuglycoside I, Ziyuglycoside II, and total saponins can promote survival of bone marrow cells through activating FAK and Erk1/2 pathways and they can also reduce suppressive cytokines MIP-2 and PF4 in BMSCs. Their ability to interact with these pathways and cytokines has never been discovered before. Our work investigates the underlining mechanisms of hematopoietic effect of saponins from *S. officinalis* L. roots for the first time and provides scientific supports to the clinical use of *S. officinalis* L. for the treatment of myelosuppression.

## Conclusion

Clinical practice in recent decades in China demonstrated that the extract of *S. officinalis* L. roots can increase WBC counts and alleviate myelotoxicity caused by anti-neoplastic therapy. However, the underlining mechanism has not been reported yet. In the present study, we evaluated and confirmed the therapeutic effect of total saponins from this plant in myelosuppressive mice. We found that total saponins and their characteristic constituents Ziyuglycoside I and Ziyuglycoside II can increase the expression of anti-apoptotic protein Mcl-1 and survivin in cytokine-depleted TF-1 cells, inhibit apoptosis of these cells and promote survival of mouse BMNCs cultured in cytokine-deprived medium through FAK and Erk1/2 activation. Furthermore, saponins-treatment reduced the levels of some suppressive cytokines both *in vitro* and *in vivo*. Our findings suggest that saponins from *S. officinalis* L. roots improve hematopoiesis by promoting survival through FAK and Erk1/2 activation and modulating cytokine production in the bone marrow. The present study provides scientific supports to the clinical use of *S. officinalis* L. roots for the treatment of myelosuppression. We hope this work will benefit the development of new therapeutic agents against myelosuppression.

## Ethics Statement

This study was carried out in accordance with the recommendations of the national guidelines, Ministry of Science and Technology of the People’s Republic of China. The protocol was approved by Animal Care and Use Committee of the Chengdu Institute of Biology.

## Author Contributions

XC and SC designed the research. XC and SC performed all the experiments. YG contributed to the analysis of *in vivo* experiments. BL, JJ, and ZW contributed to the revision of the manuscript. XC and SC wrote the paper.

## Conflict of Interest Statement

The authors declare that the research was conducted in the absence of any commercial or financial relationships that could be construed as a potential conflict of interest.

## Abbreviations

BMNCs: bone marrow nucleated cells
BMSCs: bone marrow stromal cells
DMSO: dimethyl sulfoxide
EPO: erythropoietin
Erk1/2: extracellular signal-regulated kinase ½
FAK: focal adhesion kinase
FBS: fetal bovine serum
GM-CSF: granulocyte-macrophage colony stimulating factor
G-CSF: granulocyte colony-stimulating factor
IL-3: interleukin-3
MIP-2: macrophage inflammatory protein 2
PF4: platelet factor 4
RLU: relative luminescence unit
SCF: stem cell factor
WBC: white blood cells
